# The Microbiota of Evaporative Cooling Systems and the Impact of Rainwater as an Alternative Water Source

**DOI:** 10.1002/wer.70496

**Published:** 2026-07-17

**Authors:** Nicole van Leuven, Thomas J. Tewes, Ralf Lucassen, André Lipski, Rolf Rheinschmidt, Matthias Kleinke, Dirk P. Bockmühl

**Affiliations:** ^1^ Hochschule Rhein‐Waal Kleve Germany; ^2^ Food Microbiology and Hygiene, University of Bonn Bonn Germany; ^3^ Gesundheitsamt des Kreises Mettmann Erkrath Germany

**Keywords:** biofilms, cooling tower, evaporative cooling, evaporative cooling systems, rainwater harvesting, water substitution

## Abstract

Climate change and an increasing global population are supposed to cause progressive water shortages in the future. Using rainwater in water tanks for evaporative cooling systems as a substitute might help reduce water consumption needed for cooling. Therefore, our study identified the present microbiota in evaporative cooling systems (ECS) and used an ex situ approach to identify effects of the substitution of tap water with rainwater. Culturable cell counts (CCC) based on colony‐forming units for total counts or selected pathogens, total biofilm masses based on crystal violet staining, and the bacterial composition (16S rRNA sequencing) of biofilms sampled in different ECS were considered. Data suggest that rainwater does not enhance biofilm growth, since both culturable cells and total biofilm mass tended to decrease when using rainwater instead of tap water. Interestingly, biocide treatment slightly enhanced the total biofilm mass and CCC. Sequencing data revealed differences in the microbiota when using the different water types; yet, the community shift did not include an enrichment of typical pathogens in both cases. Overall, our results suggest that a substitution of tap water with rainwater for ECS creates no disadvantage with regard to the microbial safety and might allow a reduction of financial and resource investments.

## Summary

- Biofilm formation in ECS is common and those biofilms might harbor potential pathogens.
- A suitable ex situ approach allows comparison of tap and rainwater for the use in ECS.
- Use of rainwater does not impact hygienic safety of ECS in a negative way.
- Rainwater might present a more sustainable and less costly way for operation of ECS.

## Graphical abstract

The substitution of tap water with rainwater for evaporate cooling systems creates no disadvantage with regard to the microbial safety and might allow a reduction of financial and resource investments.

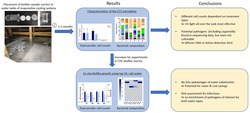

## 1 Introduction

The increasing problem of water scarcity is known worldwide, whether being due to overpopulation, water contaminations, bad management, or climate change (García‐Avila et al. 2023). A monitoring study of globally distributed wells revealed that for around 50% their groundwater levels deepen and water level declines are found more often in the early 21st century compared to the end of the 20th century (Jasechko et al. 2024). Both phenomena hint to increasing problems with water supplies in the future and suggest considering the use of rainwater as a substitution for a less resource‐consuming water usage.

In some countries rainwater is used as potable water, because it can help to reduce diseases caused by contaminated groundwater (García‐Avila et al. 2023). With less incidences of (e.g., gastrointestinal) illnesses compared to improved alternative water sources, rainwater from tanks can present a better alternative (Hamilton et al. 2019). Still, rainwater does not always meet the World Health Organization (WHO) standards for the quality of water for human consumption (García‐Avila et al. 2023). In Germany, being one of the countries where rainwater harvesting and storage is most common, it is mainly used in private settings and for nonpotable uses, for instance garden watering or toilet flushing (García‐Avila et al. 2023; Rapp et al. 2025).

Industrial settings can also be considered for the use of rainwater. Due to an increasing global population living in cities and heat problems caused by climate change, sustainable cooling systems are more utmost importance. Evaporative cooling systems (ECS) providing a less energy‐consuming alternative to compressor‐based cooling systems, are common in private as well as different industrial settings (Haile et al. 2024; Kapilan et al. 2023). There are several types of ECS (i.e., direct/indirect, two‐stage/three‐stage/multistage), which are normally chosen depending on the climate conditions and specific application. Their efficacies differ along with their energy consumption. In all systems, water is sprayed from a tank into the air by a pump and the droplets absorb heat from the air, which subsequently leads to a temperature reduction. The outside air is moved from the system by a blower or fan (Kapilan et al. 2023). When using rainwater in these types of ECS, water as well as energy can be saved. A study of 9‐years performed in three cities of Sweden showed, that for commercial buildings up to 25% of energy and 70% of water could be saved using rainwater in an indirect ECS. For residential buildings, energy savings were less. The water savings mainly depended on the cooling demand and the size of the rainwater catchment area leading to average savings of about 15% (Thomé et al. 2019).

Inside the cooling towers of ECS, temperatures between 25°C and 35°C along with constant oxygen availability and a pH‐neutral environment present favorable conditions for many microorganisms (Di Pippo et al. 2018; Liu et al. 2009). As a result, excessive biofilm formation can occur, leading to microbial corrosion, restricted heat transfer, clogging of pipes, and following energy losses, but also pose a health risk for humans (Di Pippo et al. 2018). Biofilms in ECS can be found on pipes, heat exchangers, fills and drift eliminators, and specifically on wet surfaces (VDI—The Association of German Engineers 2019). Especially members of the phyla *Pseudomonadota*, *Cyanobacteriota*, and *Acidobacteriota* have been identified in biofilms from cooling water systems (Di Gregorio et al. 2017; Wang et al. 2013), although members of other phyla have been found as well (Di Pippo et al. 2018). Because cooling towers are also known to harbor *Legionella* species, the surveillance and monitoring of this species is crucial. In particular, *Legionella pneumophila* ( *L. pneumophila* ) as the causative agent for a form of pneumonia known as Legionnaires' disease can pose a threat for human health via aerosol dispersal (Di Pippo et al. 2018). The risk of aerosol formation is given anywhere where water is being introduced into an airflow and thus is present in ECS as well. Consequently, outbreaks of legionellosis originated from ECS have been reported in the past (VDI—The Association of German Engineers 2019).

In Germany, the use of ECS and cooling towers is mainly controlled by the regulation 42nd BImSchV and extended by the code of practices VDI 2047, Part 2 for “Securing hygienically sound operation of evaporative cooling systems” (German Government 2017; VDI—The Association of German Engineers 2019). Here again, *Legionella* spp. but also *Pseudomonas aeruginosa* ( *P. aeruginosa* ) are focused on due to potential health risks. *P. aeruginosa*, as a typical organism forming biofilms, can cause different types of diseases and often shows increased resistance against biocides. Members of *Legionella* species can either cause Legionnaires' disease or Pontiac fever, an influenza‐like disease (VDI—The Association of German Engineers 2019).

Although ECS already present an energy‐efficient way to cool buildings (Haile et al. 2024), an even lower environmental impact could be achieved by substitution of tap water with rainwater. To reduce the environmental impact of cooling towers, the use of rainwater as the main water supply has already been addressed, and it has been shown that energy consumption and CO_2_‐emmission can be reduced by this means without any relevant consequences regarding corrosion and biofouling (Thomé et al. 2019). However, there is no comprehensive data on how the microbiota (including pathogenic organisms) in ESC changes when using rainwater. Moreover, due to restricted accessibility to the cooling towers and authorization issues (Di Pippo et al. 2018), the research data on the microbiota of ECS are generally limited.

Therefore, we looked at the microbiota found in ECS with different control measurements against microbial contamination. Furthermore, we developed a method to compare the impact of rainwater and tap water on ECS‐originated biofilms in an ex situ approach. Differences in culturable cell counts (CCC), general biofilm masses, and composition of biofilms were assessed. At last, the additional effect of biocide treatment for both water types was analyzed.

## 2 Methods

### 2.1 Biofilm Sampling From ECS and Sample Processing

**FIGURE 1:**
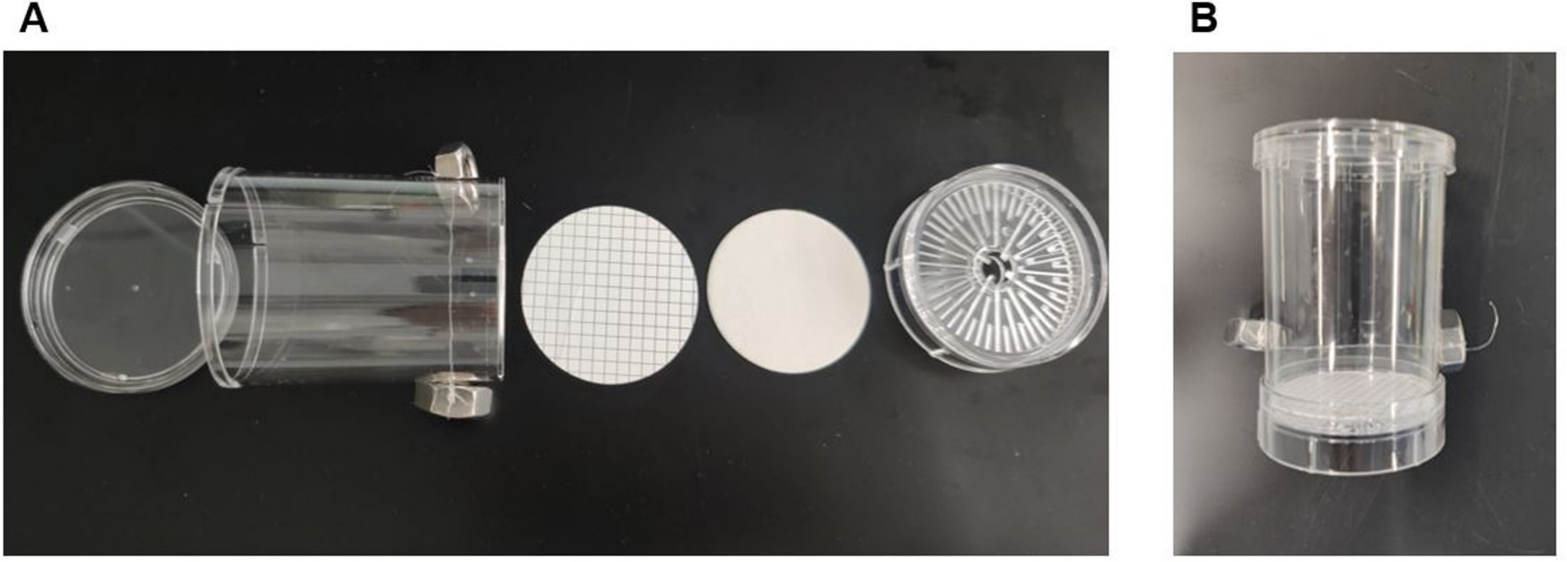
Structure of the sample carriers placed in evaporative cooling systems (ECS) disassembled (A) and assembled (B) for placement in the ECS. The filter consisted of a membrane M (with grids) and a filter layer F (without grids).

For sampling in ECS, two sterile sample carriers (Sartorius Biosart 100 Monitors; Sartorius AG, Göttingen, Germany) as shown in Figure 1 were placed in each of the monitored ECS for several weeks (Table 1). The individual components (screw nuts as weights, nylon cord) were autoclaved, and the carrier systems were assembled under sterile conditions in a fume hood. To thread the cord through the monitor, a small hole was pierced at the top and bottom using the heated tip of metal tweezers. The finished carrier systems were then packed in sterile polypropylene bags and transported in a microbiologically suitable sample case.

**TABLE 1:** Overview of sample carrier sampled from evaporative cooling systems (ECS) used for analysis.

Sample	Sample type	ECS number	Treatment	Duration of sampling
F1	Filter	1	Biocide	3 months
M1	Membrane			
F2	Filter	2	UV light (flow passage only)	1 month
M2	Membrane			
F3	Filter			
M3	Membrane			
M4	Membrane	3	UV light (total tank)	1 month
F7	Filter	4	Biocide	1 month
M7	Membrane			
F8	Filter			
M8	Membrane			

Carriers were fastened in the ECS tank with the sterile nylon thread. This setup allowed water to flow through the monitors and microorganisms to attach to the cellulose nitrate‐membrane filter.

For final sampling, carriers were removed from the ECS, transferred to the laboratory, and processed immediately. The filter consisted of the membrane (M) and a filter paper (F), and both parts were prepared separately. Due to destructed carriers and missing parts, not all duplicates and sample types could be collected (Table S1). In total, 11 samples originated in four different ECS were available for analysis (Table 1).

After transfer, filter and membrane papers were separated and each was given into a 15‐mL reaction tube containing 5‐mL Phosphate Buffered Saline (PBS; pH 7.4; Carl Roth GmbH + Co. KG, Karlsruhe, Germany). Samples were shaken at 10 min for 750 rpm at room temperature (Thermomixer comfort; Eppendorf AG, Hamburg, Germany). Afterwards, another 5 mL of PBS were added followed by vortexing (VWR VV3; VWR International Ltd., Pennsylvania, USA) for 30 s at full speed. This biofilm suspension of 10 mL was aliquoted and freeze‐protected by mixing 750 μL of sample with 750 μL 80% glycerol (≥ 99.5%; Carl Roth GmbH + Co. KG, Karlsruhe, Germany). These glycerol stocks were stored at −80°C and used for plating and DNA extraction of ECS‐biofilms as well as the inoculum for ex situ experiments.

### 2.2 Characterization of Biofilms From ECS

For each biofilm, the stock solution was plated to check the CCC in total and for bacterial groups relevant for drinking water quality (German Federal Ministry of Health 2023); 50 μL was plated in technical duplicates using a spiral plater in Log‐mode (Eddy Jet 2 W Spiral Plater; I&L Biosystems GmbH, Troisdorf, Germany). Media selection was based on different legal guidelines that are referred to in the German “Ordinance on the Quality of Water Intended for Human Consumption” (German Federal Ministry of Health 2023). Due to the sample type and low sample volume, modified and condensed protocols were performed as follows. For the total culturable cells (TCC) of aerobic culturable microorganisms, Yeast Extract Agar according to EN ISO 6222:1999 was used and incubated at 22°C for 72 h as well as 37°C for 48 h (DIN German Institute for Standardization 1999). To check for *P. aeruginosa* , Cetrimide‐Agar (PO5076A; Thermo Fisher Scientific Inc., Waltham, MA, USA) was used and incubated for 2 days at 37°C. Afterwards, colonies with blue‐green pigmentation and colonies fluorescing under UV‐light (360 ± 20 nm) were considered as *P. aeruginosa* as stated in DIN EN ISO 16266:2008 (CEN European Committee for Standardization 2008). The presence of *Legionella* spp. was checked for by plating on BCYE agar with antibiotics (PO5325A; Thermo Fisher Scientific Inc., Waltham, MA, USA) and consecutive streaking on BCYE agar with (PO5072A; Thermo Fisher Scientific Inc., Waltham, MA, USA) and without cysteine (PO5028A; Thermo Fisher Scientific Inc., Waltham, MA, USA) of *Legionella*‐suspicious colonies. Those colonies growing on cysteine‐containing agar, but not without, were considered as *Legionella* (DIN German Institute for Standardization 2019). Intestinal enterococci were detected using Slanetz and Bartley‐Agar (PO5018A; Thermo Fisher Scientific Inc., Waltham, MA, USA) and incubating for 2 days at 37°C. In case of positive results, colonies were transferred to bile aesculin azide agar (PO5062A; Thermo Fisher Scientific Inc., Waltham, MA, USA) and incubated at 44°C for 2 h. Yellow‐brownish to black colonies were then considered as positive (DIN German Institute for Standardization 2000). Finally, chromogen coliform agar (CCA; PO5318A; Thermo Fisher Scientific Inc., Waltham, MA, USA) was used to check for the presence of coliforms (pink‐red growth) and especially *Escherichia coli* (*E. coli*, blue‐purple growth). CCA‐plates were incubated at 37°C for 1 day (DIN German Institute for Standardization 2017).

Mean values of colony‐forming units (CFU) per biofilm carrier for each investigated bacterial group of duplicates were calculated and plotted.

### 2.3 Comparison of Tap and Rainwater for the Usage of ECS in an Ex Situ Biofilm Model

In order to compare biofilm growth of ECS‐originated biofilms in regular tap water and rainwater, a protocol using the CDC biofilm reactor (CBR90‐2; BioSurface Technologies Corporation, Montana, USA) was developed (Figure S1). A media supply tubing was connected to a programmable peristaltic pump, which allowed controlled media supply for the CDC reactor. The reactor itself was placed on a magnetic stir plate (IKA RH basic 2; IKA‐Werke GmbH & Co. KG, Staufen, Germany), which was additionally controlled by a time switch. As biofilm carriers, stainless steel coins (RD128‐316; BioSurface Technologies Corporation, Montana, USA) were used. As media for biofilm growth, 10% TSB dissolved in regular tap water (Location: Kleve, Germany) or rainwater was prepared and sterilized. The medium was chosen to offer standard nutrients, but promoting biofilm growth and matrix production by limiting them (Ponomareva et al. 2018). Rainwater from a tank (1 m^3^) of Rhine Waal University, Kleve, was used (Figure S2). When tapping the rainwater, the first 10 L were discarded before bottling water for media preparation. The pH of each media batch after autoclaving was measured. In all experiments, pH values of tap and rainwater‐based media did not differ more than 0.21, with rainwater‐based media showing a slightly lower pH (Table S2). Experiments with the same biofilm and treatment were performed simultaneously. Biofilms M1, M2, M4, and M7 were chosen as independent replicates for these experiments due to their different locations and treatment methods.

For initial biofilm formation, 350‐mL media was transferred to the reactor and inoculated with a 1.5‐mL stock prepared as described in 2.1. After 2 days of incubation at room temperature and without any stirring motion, the reactor was connected to media supply. For 6 days, media supply for 1 h a day followed along with simultaneous stirring at approx. 760 rpm. For a second test series, biocide (Ferrocid 4601, 25 mL/m^3^; Kurita Europe GmbH, Mannheim, Germany) was added to the reactor immediately before circulation on Day 1 and Day 4. This approach is based on information on ECS number 1, whose water tank is circulated daily and Ferrocid 4601 in the given concentration is added twice a week on average.

After 6 days (8‐days biofilm growth in total), biofilms were extracted from the coins. Rods were dipped in 50‐mL tubes containing 30‐mL PBS once to remove superficial cells. Coins were then transferred into 50‐mL tubes with conical bottom using the provided manipulation tool for the CDC reactor. In total, five coins were used for each experiment respective to a technical triplicate for CCC, one coin for DNA extraction and one coin for a crystal violet assay to compare the total biofilm masses. In case of CCC and DNA extraction, 50‐mL tubes contained 4‐mL PBS. Cells were extracted and separated following the single‐tube extraction method of ASTM E2871‐21, consisting of 3× 30s of vortexing with 2 x 30‐s sonication in‐between (ASTM International 2025). Cell suspensions were then used for either DNA extraction or CCC plating equivalent to chapter 2.2. Mean values of triplicate coins for each sample as well as means from the four independent samples were calculated.

For the crystal violet assay, the coin was transferred into 4‐mL crystal violet solution (0.1% in pure water type 1) and incubated at 30°C for 30 min. Afterwards, the coin was dipped into sterile deionized water and transferred into a fresh 50‐mL tube containing 4‐mL ethanol (min. 99.8%; AppliChem GmbH, Darmstadt, Germany). Again, biofilm extraction was done following ASTM E2817‐21 (ASTM International 2025). Photometric absorption of these cell suspensions was measured at 590 nm (BioMate 160 UV/VIS‐Spectral photometer; Thermo Fisher Scientific Inc., Waltham, MA, USA).

### 2.4 DNA Extraction and 16S rDNA Sequencing

DNA from biofilm stocks sampled from the carriers in the ECS as well as from CDC reactor samples was extracted using the FastDNA SPIN Kit for Soil (MP Biomedicals GmbH, Eschwege, Germany). One milliliter of biofilm suspension from initial stocks or the reactor experiments was centrifuged at 21.000*g* for 10 min (Heraeus Fresco 21; Thermo Fisher Scientific GmbH, Schwerte, Germany). The supernatant was discarded and the pellet resuspended in 250 μL of PBS. Instead of soil mass, the suspension was given into the matrix M tube, and the remaining extraction protocol was followed as stated by the manufacturer. Elution was done in 50‐μL DES elution solution. Sequencing of 16S (rDNA/V3V4a‐region) from DNA extracts was performed by an external service provider (Eurofins Genomics Europe Sequencing GmbH, Constance, Germany). As sequencing technology Illumina was used, Silva v138.2 acted as reference database. The raw sequencing data were filtered for high‐quality bases by trimming, quality filtering, and pruning using the software Cutadapt 2.7. After quality filtering, merging of reads was done using the software FLASH 2.2.00 with a minimum overlap of 10 bp of paired‐end reads. Statistical computing was done with the program R 4.1.3. Results were provided as abundance tables and FASTA/FASTQ files. Because some samples showed insufficient quantity and quality (Table S3), they did not pass the quality filtering for the sequencing pipeline; thus, only results for the remaining six samples are presented. Classification rates and total reads of successfully sequenced samples are shown in Table S4.

### 2.5 Data Processing and Statistical Analysis

To process data and perform statistical analysis, GraphPad Prism v. 10.6.1 (GraphPad Software Inc., San Diego, CA, USA) was used. Holm‐Šídák's multiple comparisons test was used to check for statistically significant differences of different data sets.

## 3 Results

### 3.1 Microbial Communities in ECS

**FIGURE 2:**
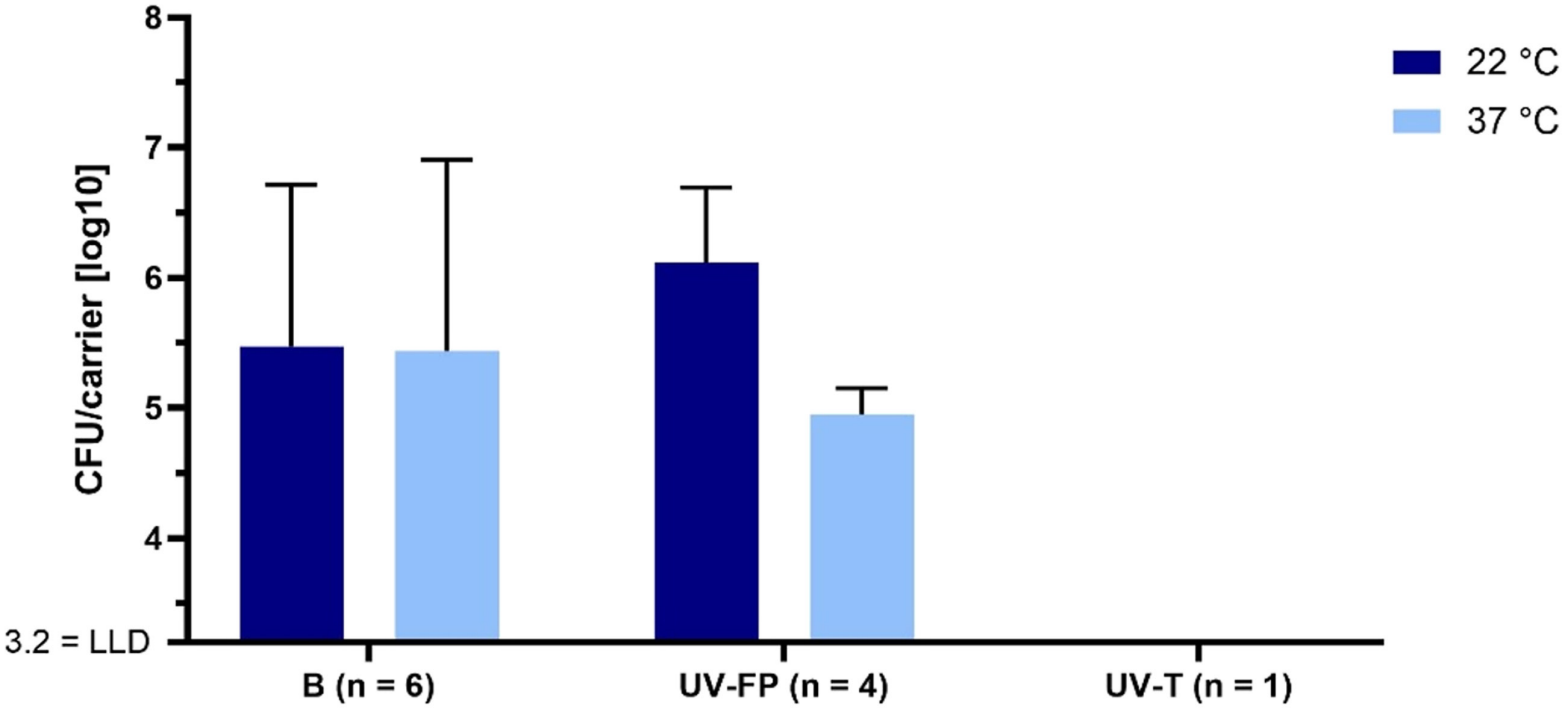
Mean total culturable cells [log10] of biofilm carriers placed in evaporative cooling systems with different treatment methods after 72 h at 22°C and 48 h at 37°C on yeast extract agar. B = Biocide treatment, UV‐FP = UV treatment (flow passage only), and UV‐T = UV treatment (tank). Bars indicate standard deviations. No statistical differences were found using Holm‐Šídák's multiple comparisons test. LLD = Lower limit of detection.

When sorting ECS samples based on their treatment method in the ECS (Figure 2), no microbial cells could be found in the sample from the UV‐treated tank. Samples from an ECS with biocide treatment showed a similar amount of culturable cells to samples from a UV passage treatment. Although slight differences were observed for different incubation temperatures, no statistical differences between incubation temperatures and treatments were found using Holm‐Šídák's multiple comparisons test.

Neither *Legionella* spp., nor coliforms, intestinal enterococci, or *P. aeruginosa* were obtained in any of the samples. Individual CCC values of sample carriers are compiled in Figure S3.

**FIGURE 3:**
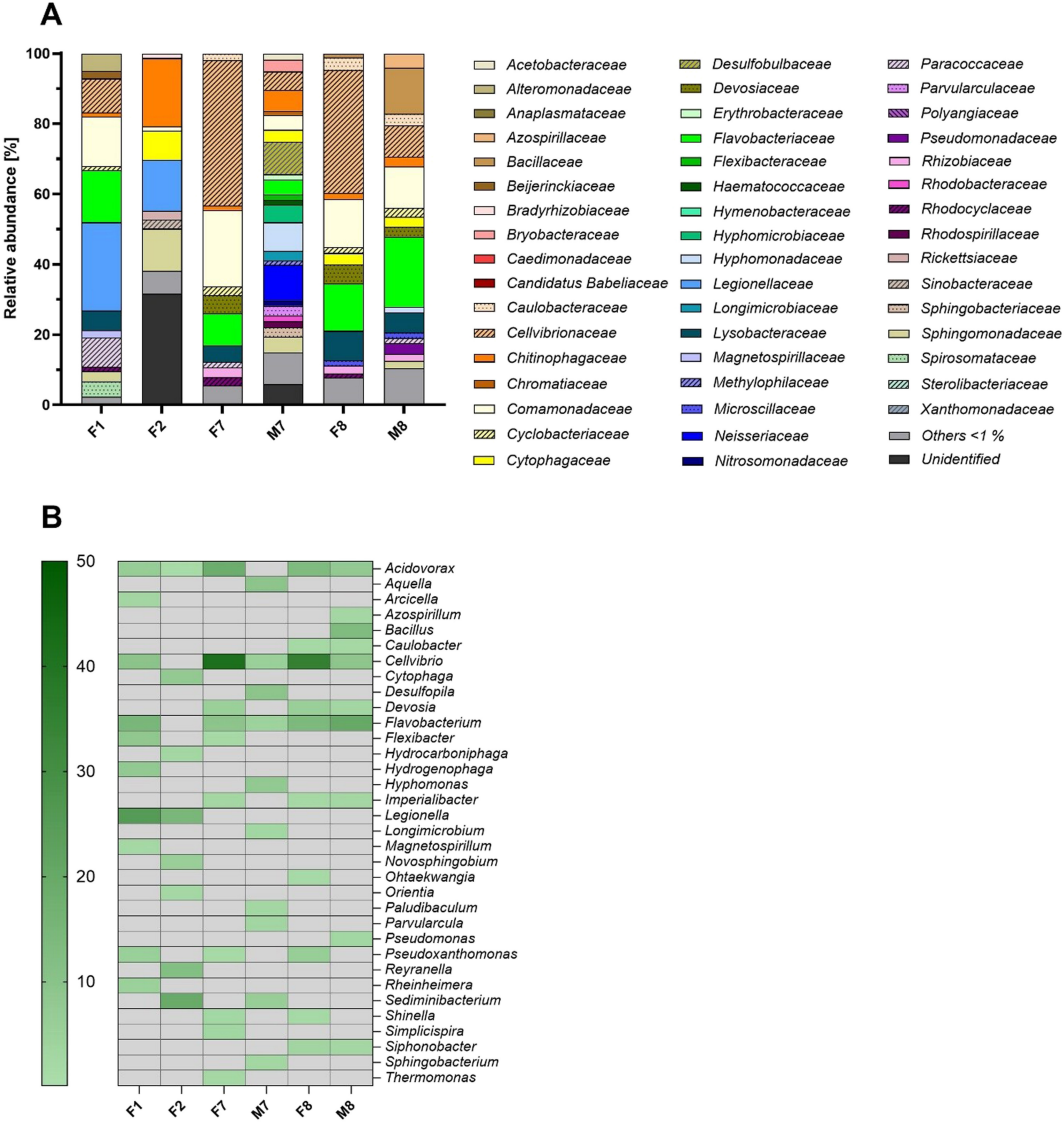
(A) Relative abundances (%) of (A) phylogenetic families identified and (B) the 10 most abundant genera in the DNA of biofilm samples originated from different evaporative cooling systems. Prevalences lower than 1.0% are summarized as “Others,” whereas “Unidentified” describes nonclassified reads at the respective phylogenetic level. F = Filter sample, M = Membrane sample. F1: biocide treatment with 3 months of sampling duration, F2: UV flow passage treatment with 1 month of sampling duration, F7/M7/F8/M8: biocide treatment with 1 month of sampling duration.

Next to culturable cells, the bacterial composition of the samples was analyzed. Here, diverse communities could be identified using 16S rRNA sequencing (Figure 3). On a family level (Figure 3A), recurring bacteria were inter alia, *Azospirillaceae*, *Cellvibrionaceae*, *Chitinophagaceae*, *Cytophagaceae*, *Flavobacteriaceae*, and *Rhizobiaceae*. For F8 and M8, the biofilms showed a similar composition independent of their source (filter or membrane). F7, which originated from the same ECS as F8 and M8, also showed big similarities especially to F8. This is supported by a lower beta‐diversity between those samples compared to other sample pairs (Figure S5). The microbiota of M7, on the other hand, was more diverse and different compared to the other samples of the same ECS (F7/M7/F8).

On genus level (Figure 3B), the genera *Acidovorax*, *Cellvibrio*, and *Flavobacterium* were found in five of six samples. However, due to the very diverse composition, most genera were present at only a low percentage. In addition, *Legionella* was detected in samples F1 and F2, whereas *Pseudomonas* was present in sample M8 only. In general, genera richness was found to be higher for membrane (M) samples than for filter (F) samples (Table S5).

### 3.2 Comparison of Tap and Rainwater for the Usage of ECS in an Ex Situ Biofilm Model

**FIGURE 4:**
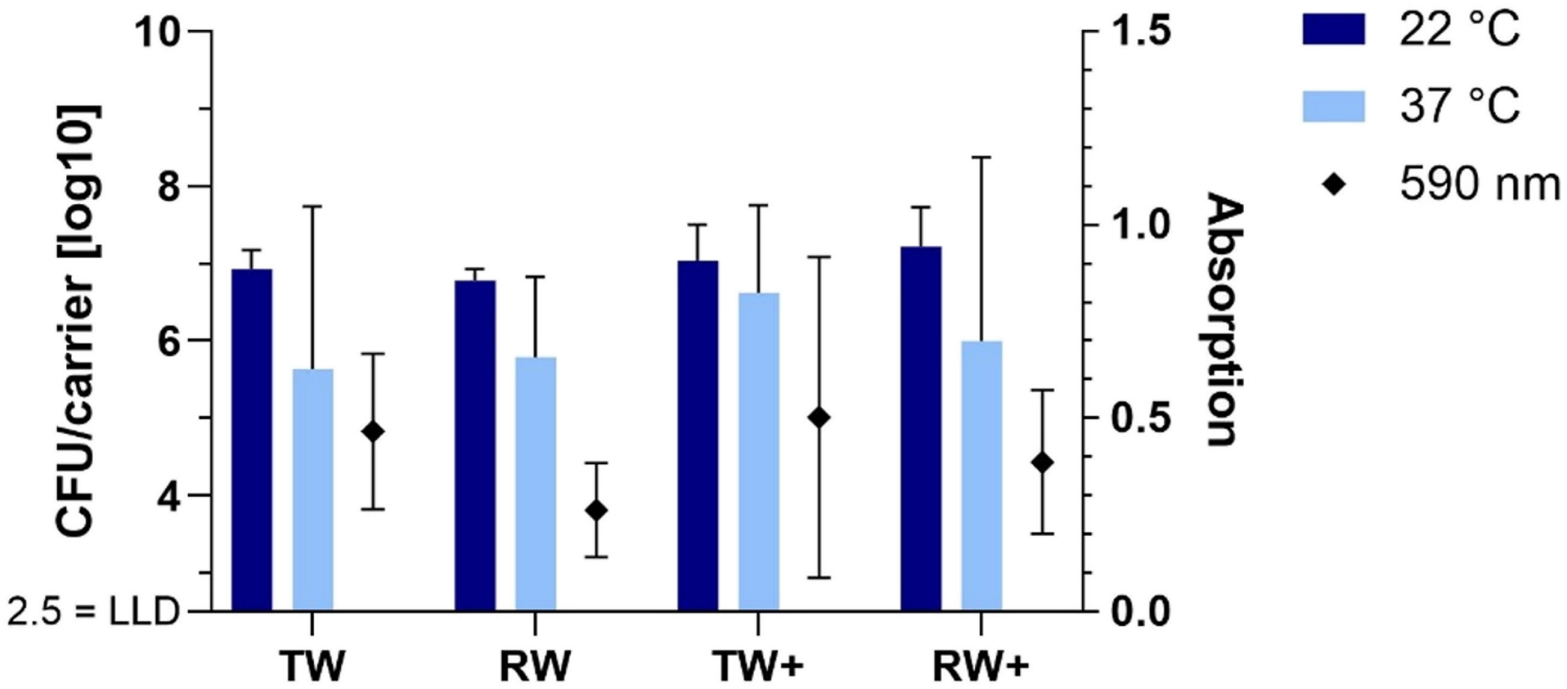
Overall means of total culturable cells (log10) of biofilm carriers placed in the CDC reactor inoculated with different ECS biofilms 72 h at 22°C and 48 h at 37°C on yeast extract agar (left *y*‐axis) and absorption of coin samples from the crystal violet assay at 590 nm (right *y*‐axis). TW = tap water, RW = rainwater, + indicates the addition of biocide. *n* = 4. No statistically significant differences were found using Holm‐Šídák's multiple comparisons test.

To reveal possible differences in biofilm growth when using tap water and rainwater as media base with and without the addition of biocide, TCC were analyzed. In addition, the biofilm mass was quantified using crystal violet staining. Neither the used water nor the treatment with biocides resulted in any statistical differences for TCC or crystal violet absorption values of the cultivated biofilms (Figure 4). Individual counts after biofilm growth in the CDC reactor for each biofilm are shown in Figure S4. However, the mean total aerobic counts at 22°C were generally higher than at 37°C, but without statistical differences. Barely any difference was found for the aerobic counts of tap and rainwater‐based experiments, whereas mean values of the crystal violet assay quantifying the total biofilm mass were tendentially higher for tap water than for rainwater, although especially for tap water with biocide treatment a high standard deviation was observed and differences were not significant.

Again, neither *Legionella* spp., nor coliforms, intestinal enterococci or *P. aeruginosa* were detected.

**FIGURE 5:**
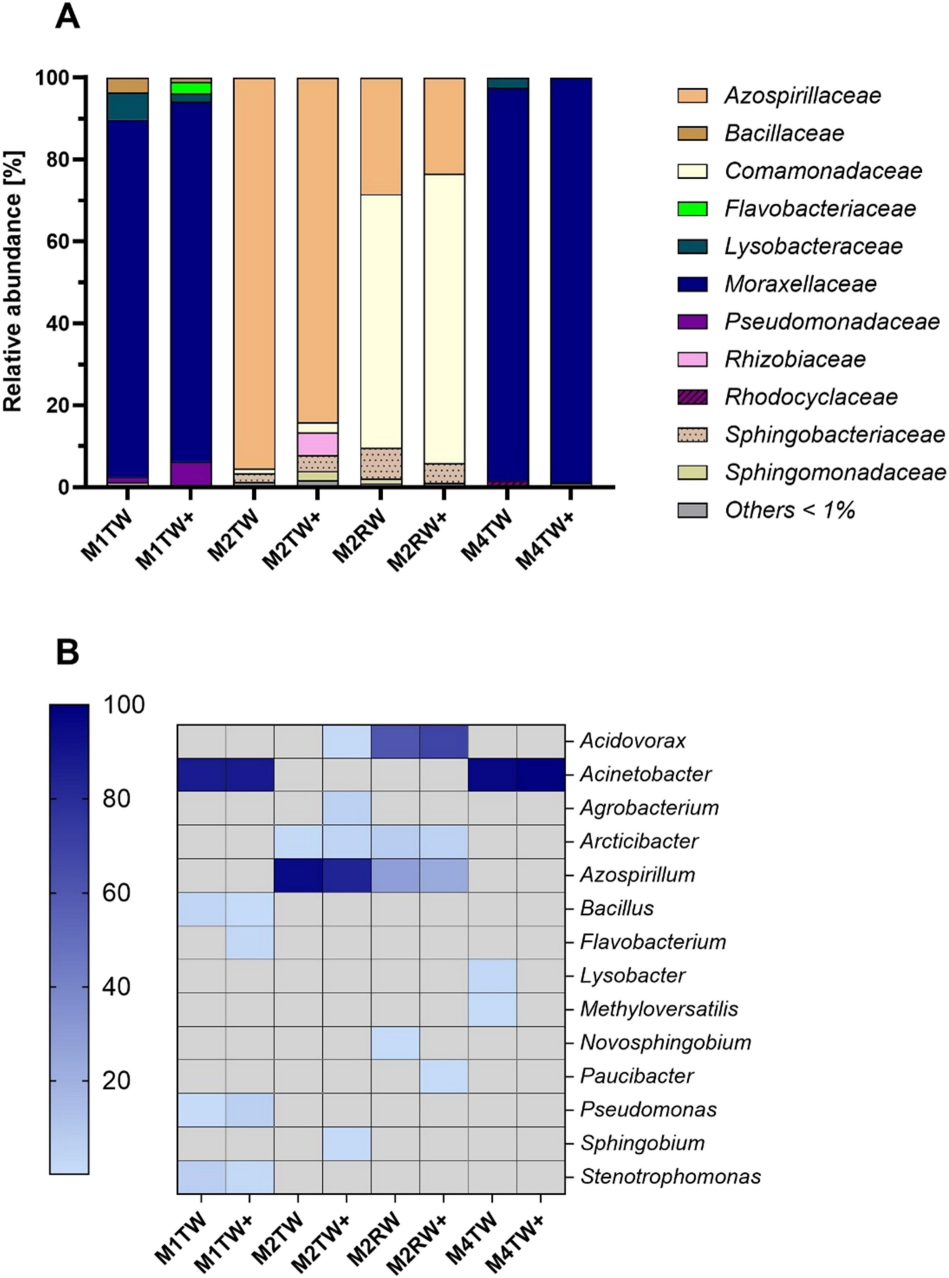
(A) Relative abundances (%) of (A) phylogenetic families identified and (B) the 10 most abundant genera in the DNA of biofilm samples grown in the CDC reactor. Prevalences lower than 1.0% are summarized as “Others.” RW = rainwater, TW = tap water, + indicates the addition of biocide. Therefore, e.g., M2TW+ = biofilm sample based on ECS biofilm sample M2, grown in tap water with the addition of biocide. M1: sampled from ECS with biocide treatment for 3 months of sampling duration, M2: sampled from ECS with UV flow passage treatment for 1 month of sampling duration, M4: sampled from ECS with UV tank treatment for 1 month of sampling duration.

Next to changes in CCC, potential changes in the microbial communities using different water types for biofilm growth (Figure 5) were analyzed. Here, for tap water samples based on M1 and M4, the family *Moraxellaceae* contributed for more than 90% of bacteria in these samples. For M2, *Azospirillaceae* was the most abundant family found when using tap water, but with the use of rainwater, *Azospirillaceae* decreased below 30%, whereas *Comamonadaceae* were enriched to above 60%. Biocide treatment led to no considerable changes in any of the investigated settings. This observation is supported by the beta‐diversity values (Figure S5).

When looking at the top 10 genera found in the samples (Figure 5B), it is likely that the presence of *Moraxellaceae* in the biofilms based on samples M1 and M4 is caused by the genus *Acinetobacter*. *Azospirillum* was the most abundant genus for M2 samples grown with tap water, whereas *Acidovorax* was more prominent in the biofilms grown in rainwater. Biofilms were mainly comprised of one or two prominent species alongside a few genera of lower abundance. In general, biofilms grown in the CDC reactor exhibited a strong decrease in alpha‐diversity compared to ECS samples (Table S5), suggesting that the number of species present in the real‐life setting is higher than in a laboratory approach.

## 4 Discussion

### 4.1 Bacterial Communities in ECS

When given the opportunity to grow on surfaces, microbiota seems to be able to reach high CCC of up to 10^8^ CFU/sample carrier in the tank water of ECS. Although we did not check for culturable cells in the water itself, a previous study suggests similar counts of up to 10^7^ CFU/mL for cooling waters (Nocker et al. 2020). For urban rainwater storages, biofilms grown on different kinds of surfaces show viable counts between 10^6^ and 10^7^ CFU/mL as well (Spinks 2007). The CCC depend on the species present and several environmental factors such as pH, water activity, and fat content in the medium. These influences, on the other hand, have an impact on heat resistance and thereby thermal inactivation rates (Spinks et al. 2006). In general, we were not able to isolate critical bacteria like *Legionella*, *Pseudomonas aeruginosa* , coliforms, or intestinal enterococci. However, *Legionella* species have been found in ECS before and proven to be sources of outbreaks (Fitzhenry et al. 2017; Nocker et al. 2020; VDI—The Association of German Engineers 2019). Temperatures found most commonly for *Legionella* isolation lie between 35°C and 45°C (World Health Organization 2007) and thereby slightly higher than temperatures in cooling towers of ECS (Di Pippo et al. 2018). Furthermore, their reproduction rates are very little below 37°C up to no proliferation below 20°C (World Health Organization 2007). From about 90 known *Legionella* species, most are known as nonpathogenic and only about 20 as human pathogens. In the case of legionellosis, the most common clinical feature is pneumonia. Independent of the causing species, pneumonia is presented very alike (Chambers et al. 2021). Especially *Legionella pneumophila* has frequently been isolated in water distribution systems, including cooling towers and evaporative condensers before in Spain, Greece, and the United States (Llewellyn et al. 2017; Mouchtouri et al. 2010; Rivera et al. 2007; Salinas et al. 2021).

Because it has been shown that molecular biological methods like detection by PCR or sequencing revealed higher prevalences than cultural methods (Llewellyn et al. 2017), this might explain our sequencing results suggesting the presence of *Legionella* without cultural confirmation in samples F1 and F2. Additionally, CFU/mL of *Legionella* found in water samples is often below the detection limit (Mouchtouri et al. 2010; Salinas et al. 2021), although for some systems they were found at higher numbers (Nocker et al. 2020). Due to the nature of our samples, especially with regard to the small sample volume, we were not able to filter a higher volume as requested by most of the guidelines for water samples and cooling towers. This led to increased lower limits of detection and also not allowed for a heat or acid pre‐treatment of our samples before the cultural detection of *Legionella* as requested by DIN EN ISO 11731:2019 (DIN German Institute for Standardization 2019). However, an additional heat or acid treatment is also known to increase the risk of *Legionella* getting into a non‐culturable status (Nocker et al. 2020). In our case, BCYE plates subsequently showed growth of accompanying microbiota that might have overgrown *Legionella* colonies, which may comprise an additional reason why *Legionella* could not be detected in culture, although DNA sequencing suggested the presence of this species in some of the samples. Still, it is also possible that the DNA fragments detected in molecular biological approaches like sequencing or qPCR are residuals of an earlier presence of viable *Legionella* cells.

One of the other mentioned bacteria to check for according to VDI 2047 is *P. aeruginosa* (VDI—The Association of German Engineers 2019). Next to *L. pneumophila* , *P. aeruginosa* is known as one of the “opportunistic premise plumbing pathogens” (Falkinham et al. 2015), which is why it would have been likely to detect it in our samples. Although there is evidence of its presence in ECS tanks, data also suggest that there is a negative correlation of the presence of *P. aeruginosa* and *Legionella* as described before (Paranjape et al. 2020). Our data confirmed that *Legionella* and *Pseudomonas* were not present in the same ECS samples. In addition, the sequencing data as well as plating on selective media indicate the absence of the potential pathogens and general hygiene indicators *Enterococcus* spp. and coliforms like *E. coli* . On the other hand, we could find genera like *Bacillus, Flavobacterium*, or non‐aeruginosa *Pseudomonas*, which can be corrosion‐associated (Rajagopal et al. 2012). Still, the community found in cooling towers depends on the structure of the tower, for example if they are closed water tanks or open to the atmosphere (Di Gregorio et al. 2017). This suggest a light‐exposure dependent development and composition of the microbial community as suggested by other studies (Di Gregorio et al. 2017; Hauer et al. 2015). For closed water tanks, Alpha‐ and Proteobacteria, especially members of *Sphingomonadaceae*, *Comamonadaceae*, and *Hyphomicrobiaceae*, are known for their presence in biofilms or in the recirculating water. *Sphingomonadaceae* are believed to be responsible for initial biofilm development (Di Gregorio et al. 2017). The microbial community might underly seasonal changes (Hauer et al. 2015; Rajagopal et al. 2012), calling for further investigations at different times of the year. Still, all three of these families could be found in our samples with *Comamonadaceae* being the most abundant. Although members of this family's type genus *Comamonas* usually show a low virulence, infections caused by *Comamonas* are reported only occasionally (Farooq et al. 2017; Ryan et al. 2022). In general, few families or genera with higher abundances were found along with many others with little abundance. This has been proves as well for rainwater tanks (Evans et al. 2009).

Four samples, originated in the same ECS, were sequenced. Although F7, M7, and F8 showed a similar composition, the microbiota M7 noticeably differed from those samples. Although most families are present in all of these four samples, the relative abundances shifted for sample M7. This might be caused by natural drifts and the natural variation by the stochastic assembly of biofilms. Both may lead to different communities even if environmental parameters are identical and the inoculum is the same (Nemergut et al. 2013; Zhou et al. 2013).

Sorting of CCC based on treatment method suggested the absence of bacterial cells in a sample from a tank with UV‐light (Figure 1). Because we were able to enrich bacteria from this sample for our biofilm growth experiments, we could conclude that there are bacteria left, but, in CCC below, our detection limit. Although there seemed to be obvious differences to samples with UV tank and biocide treatment, the differences were not statistically significant. The missing, but expected statistical differences between UV treated tanks and other treatments might probably also be caused by the small sample size for the UV light tanks (*n* = 1). Interestingly, biofilms originating from ECS with UV‐treatment as well as the ones grown from those in the CDC reactor showed less bacterial growth for the plate incubation at 37°C compared to 22°C. This might suggest a dominance of environmental bacteria over fecal contaminants, as supported by the fact that no coliforms or intestinal *Enterococci* as indicator for fecal contamination could be detected in the respective samples. For all other biofilms, plate counts for 22°C and 37°C incubation were more consistent.

Looking at the aspects discussed above, a limitation caused by the freezing step for prolonged storage and independent sample processing has to be addressed. This approach might kill bacteria and thereby decrease the amount and diversity found in the ECS samples, creating a bias for less temperature‐sensitive microorganisms. In general, cryopreservation with glycerol is commonly used and protects cells from damages caused by freezing temperatures. Yet, for some species, glycerol does not work and can even be toxic (Hubálek 2003).

### 4.2 Rainwater as an Alternative to Tap Water for ECS

The experiments performed with the CDC reactor showed that potential benefits or disadvantages of the substitution of tap water with rainwater might differ depending on the biofilm. However, data suggest in general that there is no disadvantage in using rainwater, especially with regard to total CCC of biofilms. According to VDI 2047, there is no specific value of CFU/mL of water that decides on when to introduce actions to decrease a microbial burden. Only a more than 100‐fold increase of TCC values compared to the system‐specific reference value needs to be treated by an immediate dosage of biocide and inspection of possible causes (VDI—The Association of German Engineers 2019). Consequently, because the differences between the use of tap water and rainwater do not differ that much, the substitution with rainwater seems reasonable. Even if culturable cells were higher for rainwater‐grown biofilms, a biofilm consisting of environmental microorganisms might present a neutral or even functional ecosystem with competitive properties against other pathogenic ones, leading to their exclusion (Evans et al. 2009). Furthermore, the possible formation of sludge in rainwater tanks might be beneficial for the water quality due to binding and removing contaminants (Coombes 2015; Spinks 2007).

It has to be considered that we were looking on substrate‐bound biofilms and not water samples. In our study, by using diluted media and supplying fresh media for 1 h a day, we deliberately simulated a worst case scenario for accelerated biofilm formation. For one, a generally diverse and good nutrient availability was given for a limited time. Second, following lack of nutrients promotes biofilm matrix production (Ponomareva et al. 2018). Although the results allow preliminary subsumptions, results under real conditions with lower nutrient availability might differ. Therefore, additional experiments with media compositions closer to reality are worthwhile in the future to overcome this limitation of our study.

When looking at the composition of the biofilms grown in tap and rainwater, we observed different bacterial communities independent of whether biocide was used or not. In particular, although with tap water *Azospirillaceae*, mainly represented by *Azospirillum*, outweighed, with rainwater *Acidovorax* from the family of *Comamonadaceae* was enriched. Barely any cases of human infections for both species are reported, suggesting again that substitution with rainwater is possible in ECS without increasing a potential infection risk for humans.

Most interestingly when comparing CCC of untreated and biocide treated biofilms, treated biofilms show a tendency of even higher TCC and total biofilm masses. This might suggest that the use of biocide could enhance matrix production and biofilm growth resulting in higher CCC. Especially, subinhibitory concentrations of antimicrobial substances have been proven to induce general stress responses, changes in physiology, and subsequent increasing biofilm formation before (Hemati et al. 2020; Ranieri et al. 2018). The high cell density of free‐living cells in the medium of the CDC reactor represents atypical conditions for the intended use of the biocide. Hence, an increased concentration might decrease culturable cells on biofilm coins. Additionally, organic matter from rainwater might impede the biocide's efficacy. On the other hand, it has been shown before that a reduction of detergents while using rainwater for laundry is possible due to the lower water hardness (Vargas‐Parra et al. 2019). High concentration of calcium and magnesium salts present in water with higher hardness impedes the efficacy of laundry products by precipitation with the detergents' ingredients (Smulders et al. 2002). Because the rainwater we used also had a lower water hardness (Table S6), this might apply and give the possibility of reducing the amount of needed biocide. As the biocide did not reduce TCC values, there was no advantage in using it for both types of water. The code of practice VDI 2047 states that the use of biocides should be refrained from if not necessary (VDI—The Association of German Engineers 2019), which would be somehow supported by our experiments.

The use of rainwater for cooling towers has great potential to save water, and the economic advantage is believed to increase due to rising prices for water from other resources (Thomé et al. 2019). Next to environmental aspects, economic advantages can be achieved by considering rainwater to (partly) substitute tap water. The specific financial benefit will, of course, depend on the current water price, the capacity of the rainwater tank, and the type of building (Morales‐Pinzón et al. 2015). The code of practice does not dictate which water to use as cooling water, as long as the raw water is processed as needed before it is used as the make‐up water to substitute water loss of the cooling water (VDI—The Association of German Engineers 2019). Therefore, rainwater does present a realistic substitute for tap water in ECS.

But some challenges remain after rainwater harvesting including its treatment before usage. Requirements to protect pipes and equipment from erosion, plugging, and fouling are waters with low turbidity, low hardness, and a suitable pH value (Zhao and Xu 2012). Therefore, organic matter found in rainwater might favor these factors. Still, (partly) substitution of rainwater passes corrosion tests for different materials based on ASTM 2688 (Thomé et al. 2019). A pH value > 7 is used in most industrial cooling water systems to decrease corrosion by alkalinity (Rajagopal et al. 2012). Our media's pH value was only slightly higher than 7 (Table S2), so that an adjustment of it to a more alkaline milieu in future experiments helps to modify our method even closer to reality.

## 5 Conclusion

In our experiments, we were able to characterize the microbiota found in diverse ECS with different disinfection strategies. Disinfection with UV lamps placed in the tank proved to show less biofilm growth on our carriers compared to UV treatment only in flow passages or biocide treatment. Pathogens of interest could not be identified in culture, although sequencing data suggest their presence in some cases.

Our data in general support substituting tap water with rainwater for ECS, providing a sustainable and less cost intensive way for cooling systems of buildings. Similar CCC were obtained independent of the used water type, and we could not observe an advantage in adding biocide. Although differences in the biofilms based on tap and rainwater were seen, no enrichment of critical or harmful genera took place. Still, the widely varying properties of rainwater (e.g., pH value or the presence of organic substances) have to be considered.

Taken together, our study provided first insights of the effects of the water type on biofilm formation in ECS. To gain deeper knowledge, the protocol might be further modified by changing incubation parameters or extended by adding additional substances like corrosion protection or defoamer that could influence the microbiota in ECS.

## Author Contributions

**Nicole van Leuven:** investigation, writing – original draft. **Thomas J. Tewes:** investigation, methodology. **Ralf Lucassen:** conceptualization, validation. **André Lipski:** supervision. **Rolf Rheinschmidt:** conceptualization, validation. **Matthias Kleinke:** funding acquisition, project administration, writing – review and editing. **Dirk P. Bockmühl:** conceptualization, funding acquisition, writing – review and editing, project administration, supervision.

## Ethics Statement

The authors have nothing to report.

## Conflicts of Interest

The authors declare no conflicts of interest.

## Supporting information


**Table S1:** Overview of samples available for cell extraction and 16S rRNA sequencing. Grayed samples are not considered in the manuscript at all due to missing samples.
**Figure S1:** CDC reactor setup for comparing the biofilm growth of ECS‐biofilms in tap water and rainwater.
**Figure S2:** Rainwater tank (1 m^3^) located at Rhine‐Waal University of Applied Sciences in Kleve.
**Table S2:** pH values of media used for CDC reactor experiments prepared in tap water (TW) or rainwater (RW) without and with (+) addition of biocide during biofilm growth.
**Table S3:** Overview and properties of samples that did not pass the quality control for 16S rRNA sequencing (V3V4a region).
**Table S4:** Read classification rates of successfully sequenced samples.
**Figure S3:** Total viable cell counts [log10] of biofilm carriers placed in evaporative cooling systems after 72 h at 22°C and 48 h at 37°C on yeast extract agar. Bars indicate standard deviations (*n* = 2), the compact letter display indicates statistical differences using Tukey's multiple comparisons test. LLD = Lower limit of detection.
**Figure S4:** Total viable cell counts [log10] of biofilm carriers placed in the CDC reactor inoculated with different ECS biofilms 72 h at 22°C and 48 h at 37°C on yeast extract agar (left *y*‐axis) and absorption of coin samples from the crystal violet assay at 590 nm (right *y*‐axis). (A) Biofilm M1, (B) Biofilm M2, (C) Biofilm M4, and (D) Biofilm M7. TW = tap water, RW = rainwater, + indicates the addition of biocide. Bars indicate standard deviations (*n* = 3) Asterisks implicate statistical significances (* = *p* ≤ 0.05, ** = *p* ≤ 0.01, *** = *p* ≤ 0.001, **** = *p* ≤ 0.0001) using Šídák's multiple comparisons test to compare water and treatment types.
**Table S5:** Different Alpha‐diversity measurements and their means for sequenced samples. Samples originated in evaporative cooling systems (ECS) or grown in the CDC reactor. TW = tap water, RW = rainwater, + indicates the addition of biocide.
**Figure S5:** Beta‐Diversities between samples originated in evaporative cooling systems and biofilms grown in the CDC biofilm reactor. TW = tap water, RW = rainwater, + indicates the addition of biocide.
**Table S6:** Water hardness (°dH) of water samples used for CDC reactor experiments. TW = tap water, RW = rainwater. Latest official info (16.10.2024) on water hardness in Kleve, Germany, is 6.59°dH (https://www.stadtwerke‐kleve.de/privatkunden/wasser/wasserqualitaet‐1).

## Data Availability

The data that support the findings of this study are openly available in Zenodo at https://zenodo.org/, reference number 10.5281/zenodo.19222432.
