# Supplementary material for: The Microbiota of Evaporative Cooling Systems and the Impact of Rainwater as an Alternative Water Source
Source: Water Environ Res. 2026 Jul 17;98(7):e70496. doi: 10.1002/wer.70496 (PMC13377001; doi:10.1002/wer.70496)
Supplement: Supplementary file 1 — Table S1: Overview of samples available for cell extraction and 16S rRNA sequencing. Grayed samples are not considered in the manuscript at all due to missing samples. Figure S1: CDC reactor setup for comparing the biofilm growth of ECS‐biofilms in tap water and rainwater. Figure S2: Rainwater tank (1 m3) located at Rhine‐Waal University of Applied Sciences in Kleve. Table S2: pH values of media used for CDC reactor experiments prepared in tap water (TW) or rainwater (RW) without and with (+) addition of biocide during biofilm growth. Table S3: Overview and properties of samples that did not pass the quality control for 16S rRNA sequencing (V3V4a region). Table S4: Read classification rates of successfully sequenced samples. Figure S3: Total viable cell counts [log10] of biofilm carriers placed in evaporative cooling systems after 72 h at 22°C and 48 h at 37°C on yeast extract agar. Bars indicate standard deviations (n = 2), the compact letter display indicates statistical differences using Tukey's multiple comparisons test. LLD = Lower limit of detection. Figure S4: Total viable cell counts [log10] of biofilm carriers placed in the CDC reactor inoculated with different ECS biofilms 72 h at 22°C and 48 h at 37°C on yeast extract agar (left y‐axis) and absorption of coin samples from the crystal violet assay at 590 nm (right y‐axis). (A) Biofilm M1, (B) Biofilm M2, (C) Biofilm M4, and (D) Biofilm M7. TW = tap water, RW = rainwater, + indicates the addition of biocide. Bars indicate standard deviations (n = 3) Asterisks implicate statistical significances (* = p ≤ 0.05, ** = p ≤ 0.01, *** = p ≤ 0.001, **** = p ≤ 0.0001) using Šídák's multiple comparisons test to compare water and treatment types. Table S5: Different Alpha‐diversity measurements and their means for sequenced samples. Samples originated in evaporative cooling systems (ECS) or grown in the CDC reactor. TW = tap water, RW = rainwater, + indicates the addition of biocide. Figure S5: Beta‐Diversities betwee [file WER-98-e70496-s001.docx]

**Table S1:** Overview of samples available for cell extraction and 16S rRNA sequencing. Greyed samples are not considered in the manuscript at all due to missing samples.

| **ECS number** | **Treatment** | **Duration of sampling** | **Sample** | **Cell extraction** | **Sequencing** |
| --- | --- | --- | --- | --- | --- |
| 1 | Biocide | 3 months | F1 | 🗸 | 🗸 |
|  |  |  | M1 | 🗸 | 🗴 |
| 2 | UV light (flow passage only) | 1 month | F2 | 🗸 | 🗸 |
|  |  |  | M2 | 🗸 | 🗴 |
|  |  |  | F3 | 🗸 | 🗴 |
|  |  |  | M3 | 🗸 | 🗴 |
| 3 | UV light (total tank) | 1 month | M4 | 🗸 | 🗴 |
|  |  |  | F4 | 🗴 | 🗴 |
|  |  |  | M5 | 🗴 | 🗴 |
|  |  |  | F5 | 🗴 | 🗴 |
| 4 | Biocide | 1 month | F7 | 🗸 | 🗸 |
|  |  |  | M7 | 🗸 | 🗸 |
|  |  |  | F8 | 🗸 | 🗸 |
|  |  |  | M8 | 🗸 | 🗸 |


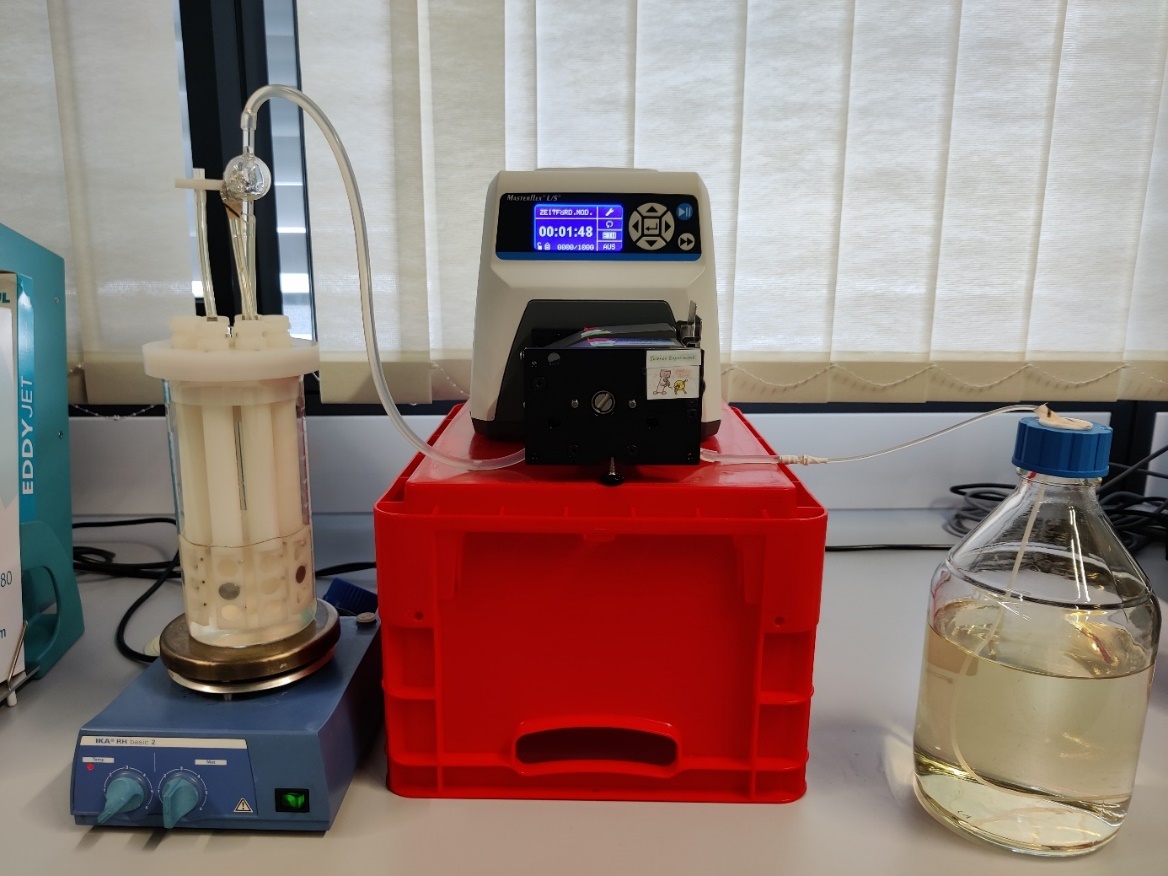


**Figure S1:** CDC reactor setup for comparing the biofilm growth of ECS-biofilms in tap water and rain water.


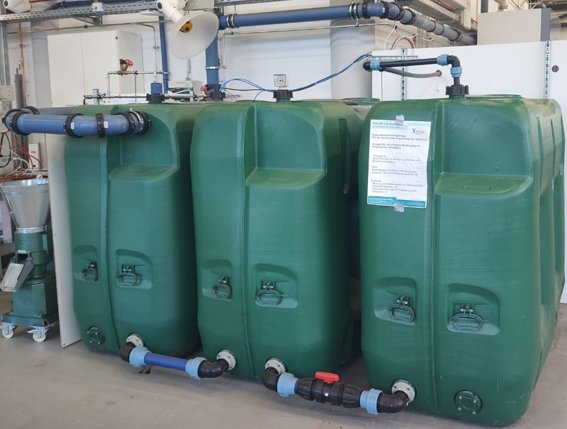


**Figure S2:** Rain water tank (1 m^3^) located at Rhine-Waal University of Applied Sciences in Kleve.

**Table S2:** pH values of media used for CDC reactor experiments prepared in tap water (TW) or rain water (RW) without and with (+) addition of biocide during biofilm growth.

| **Experiment** | **10% TSB in TW** | **10% TSB in RW** |
| --- | --- | --- |
| M1 TW vs. RW | 7.37 | 7.33 |
| M2 TW vs. RW | 7.60 | 7.40 |
| M4 TW vs. RW | 7.52 | 7.40 |
| M7 TW vs. RW | 7.57 | 7.45 |
| M1 TW+ vs. RW+ | 7.62 | 7.43 |
| M2 TW+ vs. RW+ | 7.55 | 7.44 |
| M4 TW+ vs. RW+ | 7.48 | 7.31 |
| M7 TW+ vs. RW+ | 7.52 | 7.40 |

**Table S3:** Overview and properties of samples that did not pass the quality control for 16S rRNA sequencing (V3V4a region)

| **Sample Name** | **Target** | **Library QC status** | **Library molarity [nmol/L]** | **Library QC problem** |
| --- | --- | --- | --- | --- |
| M1 | V3V4a | failed | 0.47 | 2nd QC conc. too low |
| M2 | V3V4a | failed | 0.48 | 2nd QC conc. too low |
| F2 | V3V4a | failed | 0.58 | 2nd QC conc. too low |
| M3 | V3V4a | failed | 0.5 | 2nd QC conc. too low |
| F3 | V3V4a | failed | 0.58 | 2nd QC conc. too low |
| M4 | V3V4a | failed | 0.56 | 2nd QC conc. too low |
| M7 | V3V4a | failed | 0.81 | 2nd QC conc. too low |
| M1RW | V3V4a | failed | 1.22 | 2nd QC conc. too low |
| M4RW | V3V4a | failed | 0.58 | 2nd QC conc. too low |
| M7TW | V3V4a | failed | 0.94 | 2nd QC conc. too low |
| M7RW | V3V4a | failed | 0.57 | 2nd QC conc. too low |
| M1RWB | V3V4a | failed | 0.68 | 2nd QC conc. too low |
| M4RWB | V3V4a | failed | 1.18 | 2nd QC conc. too low |
| M7TWB | V3V4a | failed | 0.46 | 2nd QC conc. too low |
| M7RWB | V3V4a | failed | 0.83 | 2nd QC conc. too low |

**Table S4:** Read classification rates of successfully sequenced samples.

| Read classification rate | | | | | |
| --- | --- | --- | --- | --- | --- |
| **Sample** | **Total_Reads** | **Total_Classified** | **Ambiguous** | **Total_Unclassified** | **Kingdom** |
| F1 | 71.73K | 74,40% | 15,30% | 10,30% | 100,00% |
| F7 | 71.02K | 89,00% | 10,70% | 0,30% | 100,00% |
| F8 | 71.38K | 91,40% | 8,30% | 0,30% | 100,00% |
| M8 | 70.88K | 78,90% | 18,50% | 2,60% | 100,00% |
| M1TW | 73.34K | 100,00% | 0,00% | 0,00% | 100,00% |
| M1TW+ | 73.36K | 100,00% | 0,00% | 0,00% | 100,00% |
| M2RW | 71.79K | 100,00% | 0,00% | 0,00% | 100,00% |
| M2RW+ | 71.73K | 100,00% | 0,00% | 0,00% | 100,00% |
| M2TW | 73.81K | 100,00% | 0,00% | 0,00% | 100,00% |
| M2TW+ | 73.31K | 100,00% | 0,00% | 0,00% | 100,00% |
| M4TW | 73.66K | 100,00% | 0,00% | 0,00% | 100,00% |
| M4TW+ | 73.91K | 100,00% | 0,00% | 0,00% | 100,00% |
|  |  |  |  |  |  |
|  | Taxonomic level wise classification rate | | | | |
| **Sample** | **Phylum** | **Class** | **Order** | **Family** | **Genus** |
| F1 | 100,00% | 100,00% | 89,87% | 89,67% | 74,41% |
| F7 | 100,00% | 100,00% | 99,96% | 99,66% | 89,00% |
| F8 | 100,00% | 100,00% | 99,97% | 99,68% | 91,38% |
| M8 | 100,00% | 99,98% | 99,73% | 97,37% | 78,87% |
| M1TW | 99,99% | 99,99% | 99,99% | 99,99% | 99,99% |
| M1TW+ | 100,00% | 100,00% | 100,00% | 100,00% | 100,00% |
| M2RW | 100,00% | 100,00% | 100,00% | 100,00% | 100,00% |
| M2RW+ | 99,99% | 99,99% | 99,99% | 99,99% | 99,99% |
| M2TW | 99,99% | 99,99% | 99,99% | 99,99% | 99,99% |
| M2TW+ | 99,99% | 99,99% | 99,99% | 99,99% | 99,99% |
| M4TW | 99,99% | 99,99% | 99,99% | 99,99% | 99,99% |
| M4TW+ | 99,99% | 99,99% | 99,99% | 99,99% | 99,99% |


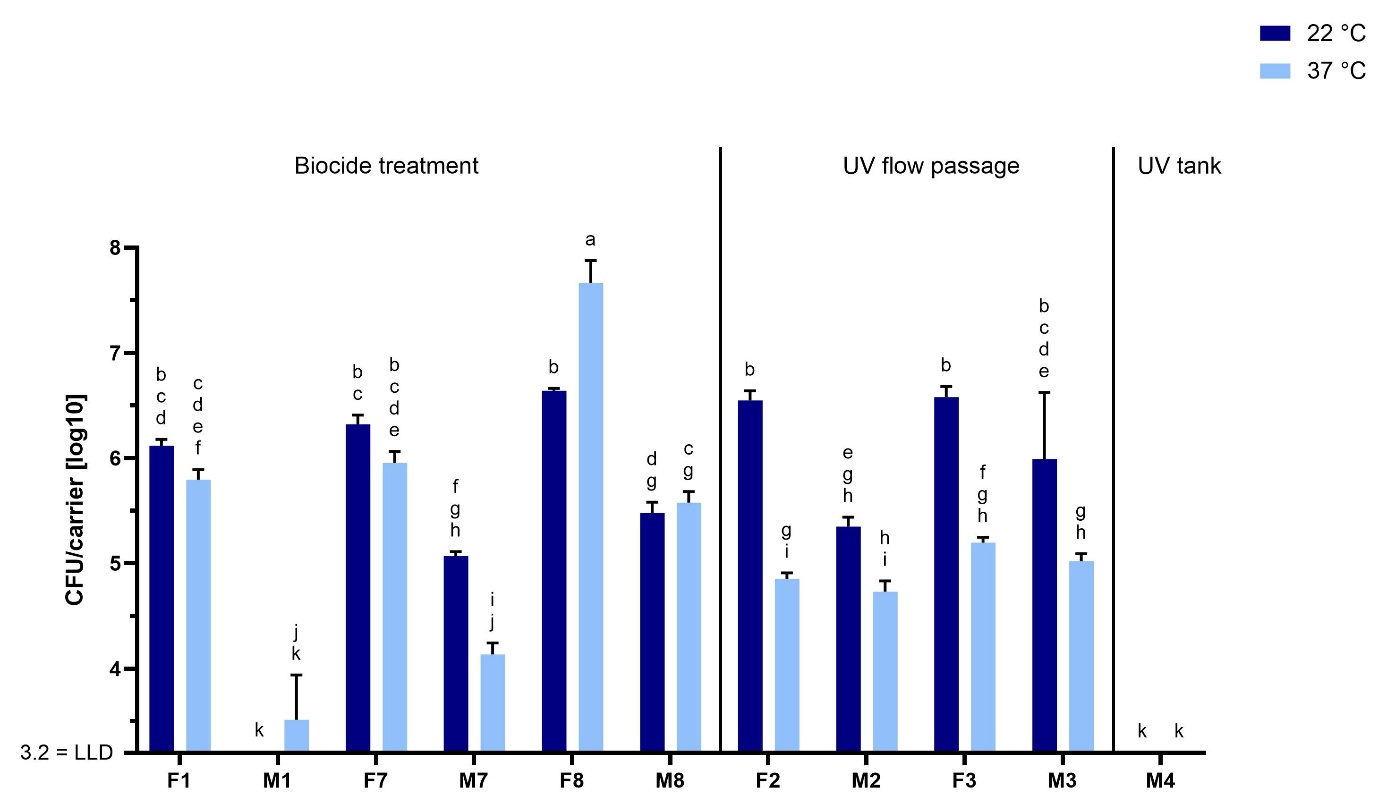


**Figure S3:** Total viable cell counts [log10] of biofilm carriers placed in evaporative cooling systems after 72 h at 22 °C and 48 h at 37 °C on yeast extract agar. Bars indicate standard deviations (n = 2), the compact letter display indicates statistical differences using Tukey's multiple comparisons test. LLD = Lower limit of detection.


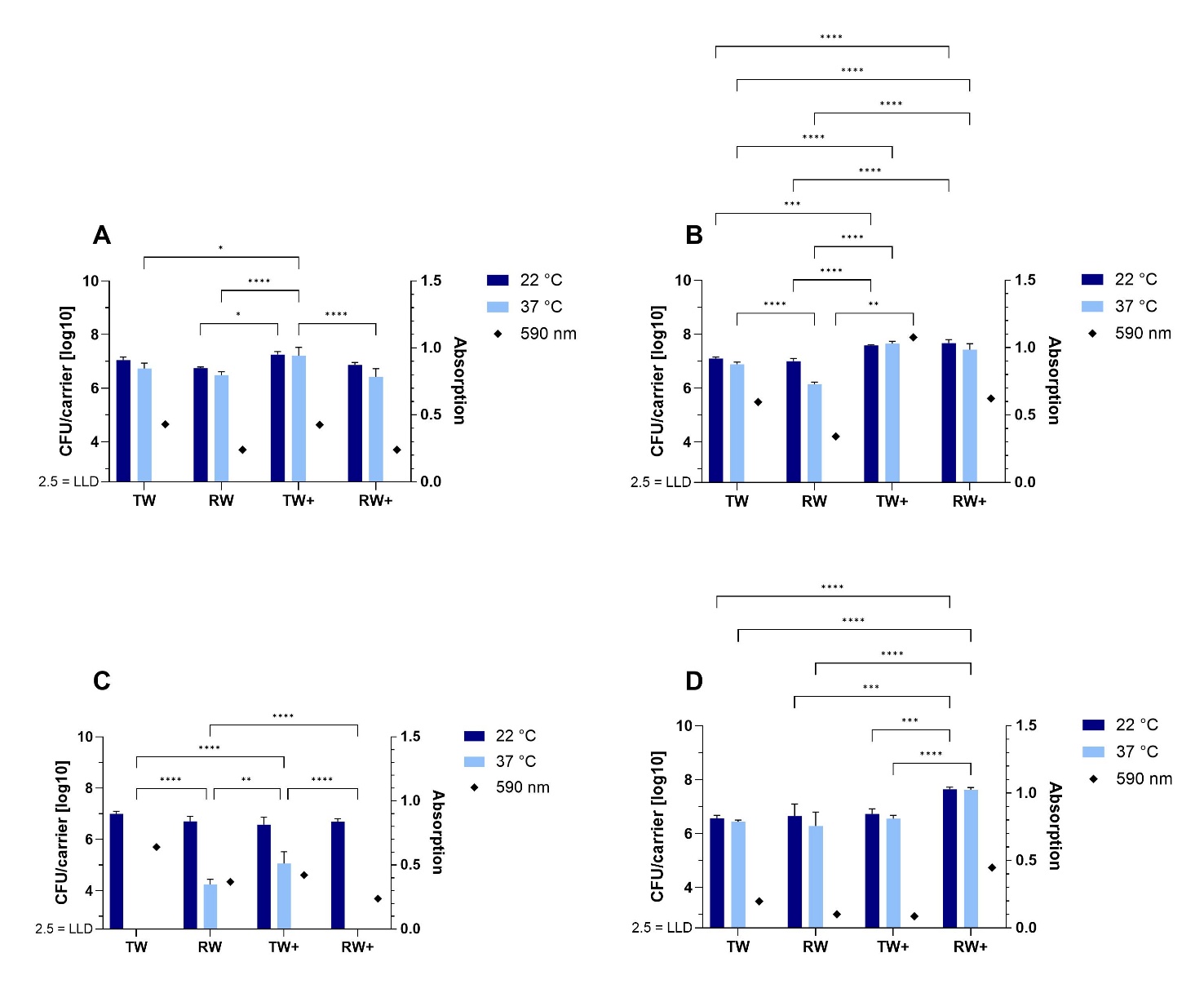


**Figure S4:** Total viable cell counts [log10] of biofilm carriers placed in the CDC reactor inoculated with different ECS biofilms 72 h at 22 °C and 48 h at 37 °C on yeast extract agar (left y-axis) and absorption of coin samples from the crystal violet assay at 590 nm (right y-axis). A) Biofilm M1, B) Biofilm M2, C) Biofilm M4, D) Biofilm M7. TW = tap water, RW = rain water, + indicates the addition of biocide. Bars indicate standard deviations (n = 3) Asterisks implicate statistical significances (* = p ≤ 0.05, ** = p ≤ 0.01, *** = p ≤ 0.001, **** = p ≤ 0.0001) using Šídák's multiple comparisons test to compare water and treatment types.

**Table S5:** Different Alpha-diversity measurements and their means for sequenced samples. Samples originated in evaporative cooling systems (ECS) or grown in the CDC reactor. TW = tap water, RW = rain water, + indicates the addition of biocide.

| **Sample** | **Sample Type** | **Water type** | **Shannon** | **Simpson** | **Chao1** | **InvSimpson** | **Evenness** | **Richness** | **FishersAlpha** |
| --- | --- | --- | --- | --- | --- | --- | --- | --- | --- |
| F1 | ECS | n. a. | 2,58 | 0,88 | 66 | 8,43 | 0,62 | 66 | 7,48 |
| F7 | ECS | n. a. | 2,22 | 0,78 | 69 | 4,54 | 0,52 | 69 | 7,65 |
| F8 | ECS | n. a. | 2,52 | 0,83 | 83 | 6 | 0,57 | 83 | 9,38 |
| M8 | ECS | n. a. | 3,32 | 0,92 | 132 | 12,94 | 0,68 | 132 | 16,21 |
| M1TW | CDC reactor | Tap water | 0,56 | 0,24 | 23 | 1,31 | 0,18 | 23 | 2,21 |
| M1TW+ | CDC reactor | Tap water | 0,54 | 0,22 | 17 | 1,29 | 0,19 | 17 | 1,58 |
| M2RW | CDC reactor | Rain water | 1,02 | 0,54 | 22 | 2,18 | 0,33 | 22 | 2,11 |
| M2RW+ | CDC reactor | Rain water | 0,87 | 0,46 | 17 | 1,86 | 0,31 | 17 | 1,59 |
| M2TW | CDC reactor | Tap water | 0,27 | 0,09 | 22 | 1,1 | 0,09 | 22 | 2,1 |
| M2TW+ | CDC reactor | Tap water | 0,74 | 0,29 | 28 | 1,4 | 0,22 | 28 | 2,75 |
| M4TW | CDC reactor | Tap water | 0,21 | 0,08 | 14 | 1,08 | 0,08 | 14 | 1,28 |
| M4TW+ | CDC reactor | Tap water | 0,07 | 0,02 | 6 | 1,02 | 0,04 | 6 | 0,5 |
|  | | | | | | | | | |
|  |  | **Mean ECS samples (n = 4)** | 2,66 | 0,8525 | 87,5 | 7,9775 | 0,5975 | 87,5 | 10,18 |
|  |  | **Mean CDC reactor samples (n = 8)** | 0,535 | 0,2425 | 18,625 | 1,405 | 0,18 | 18,625 | 1,765 |
|  |  | **Mean TW CDC samples (n = 6)** | 0,40 | 0,16 | 18,33 | 1,20 | 0,13 | 18,33 | 1,74 |
|  |  | **Mean RW CDC samples (n = 2)** | 0,945 | 0,5 | 19,5 | 2,02 | 0,32 | 19,5 | 1,85 |


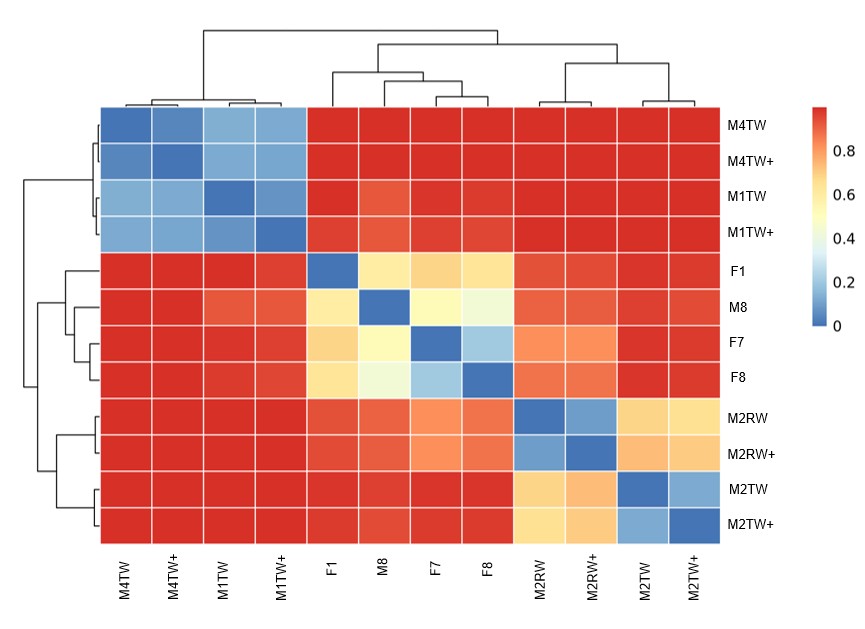


**Figure S5:** Beta-Diversities between samples originated in evaporative cooling systems and biofilms grown in the CDC biofilm reactor. TW = tap water, RW = rain water, + indicates the addition of biocide.

**Table S6:** Water hardness (°dH) of water samples used for CDC reactor experiments. TW = tap water, RW = rain water. Latest official info (16.10.2024) on water hardness in Kleve, Germany, is 6.59 °dH (https://www.stadtwerke-kleve.de/privatkunden/wasser/wasserqualitaet-1)

| **Experiment** | **TW** | **RW** |
| --- | --- | --- |
| M1 TW vs. RW | 6 | 1 |
| M2 TW vs. RW | 7 | 1 |
| M4 TW vs. RW | 6-7 | 1 |
| M7 TW vs. RW | 3 | 1 |
| M1 TW+ vs. RW+ | 3 | 1 |
| M2 TW+ vs. RW+ | 7 | 1 |
| M4 TW+ vs. RW+ | 5 | 1 |
| M7 TW+ vs. RW+ | 6 | 1 |
